# Case Report: Compound Heterozygous Variants in *MOCS3* Identified in a Chinese Infant With Molybdenum Cofactor Deficiency

**DOI:** 10.3389/fgene.2021.651878

**Published:** 2021-04-08

**Authors:** Qi Tian, Yang Cao, Li Shu, Yongjun Chen, Ying Peng, Yaqin Wang, Yuanyuan Chen, Hua Wang, Xiao Mao

**Affiliations:** ^1^Department of Medical Genetics, Maternal and Child Health Hospital of Hunan Province, Changsha, China; ^2^Department of Radiology, Chenzhou First People's Hospital, Chenzhou, China; ^3^National Health Commission Key Laboratory of Birth Defects Research, Prevention and Treatment, Hunan Provincial Maternal and Child Health Care Hospital, Changsha, China; ^4^Department of School of Life Sciences, Central South University, Changsha, China; ^5^Department of Neurology, Nanhua Affiliated Hospital, University of South China, Hengyang, China; ^6^Health Management Center, The Third Xiangya Hospital, Central South University, Changsha, China; ^7^Reproductive Center of Maternal and Child Health Hospital of Hunan Province, Changsha, China

**Keywords:** *MOCS3*, neurodevelopmental outcome, sulfite oxidase, whole exome sequencing, molybdenum cofactor deficiency

## Abstract

**Background:** The molybdenum cofactor (Moco) deficiency in humans results in the inactivity of molybdenum-dependent enzymes and is caused by pathogenic variants in *MOCS1* (*Molybdenum cofactor synthesis 1*), *MOCS2* (*Molybdenum cofactor synthesis 2*), and *GPHN* (*Gephyrin*). These genes along with *MOCS3* (*Molybdenum cofactor synthesis 3*) are involved in Moco biosynthesis and providing cofactors to Moco-dependent enzymes. Until now, there was no study to confirm that *MOCS3* is a causative gene of Moco deficiency.

**Methods:** Detailed clinical information was collected in the pedigree. The Whole-exome sequencing (WES) accompanied with Sanger sequencing validation were performed.

**Results:** We described the clinical presentations of an infant, born to a non-consanguineous healthy family, diagnosed as having *MOCS3* variants caused Moco deficiency and showing typical features of Moco deficiency including severe neurologic symptoms and cystic encephalomalacia in the brain MRI, resulting in neonatal death. Compound heterozygous variants in the *MOCS3* gene were identified by WES. Positive sulfite and decreased levels of uric acid in plasma and urine were detected.

**Conclusion:** To our knowledge, this is the first case of *MOCS3* variants causing Moco deficiency. Our study may contribute to genetic diagnosis of Moco deficiency and future genetic counseling.

## Introduction

The molybdenum cofactor (Moco) deficiency in humans results in the inactivity of molybdenum-dependent enzymes including sulfite oxidase, xanthine oxidoreductase, and aldehyde oxidase (Mayr et al., 2021). The inactivation of these enzymes in neurons was related to severe neurological symptoms and residual diseases (Zaki et al., 2016). The etiology of Moco deficiency was caused by pathogenic variants in *MOCS1* (*Molybdenum cofactor synthesis 1*), *MOCS2* (*Molybdenum cofactor synthesis 2*), and *GPHN* (*Gephyrin*). The typical clinical features of Moco deficiency patients with these variants are characterized by neonatal-onset intractable seizures, followed by feeding difficulty, developmental delay, and neonatal deaths (Mechler et al., 2015).

The signs and symptoms of patients with Moco deficiency are attributed to the pathogenesis of inactivating the molybdenum-dependent enzymes (Atwal and Scaglia, 2016). The enzyme inactivation subsequently leads to the accumulation of metabolites including sulfite, xanthine, hypoxanthine, etc. It is believed that the pathogenesis is caused by accumulated metabolites, and it may exert toxic effects on neurons and lead to seizures, encephalopathy, and other neurological symptoms (Mechler et al., 2015).

Until now, there have been no cases reported with the *MOCS3 (Molybdenum cofactor synthesis 3)* variation that presented with the typical features of Moco deficiency. The only reported case related to *MOCS3* showed a slight change in sulfite metabolites, and as a result the patient only presented mild neurologic disorder symptoms such as high muscular tone and limited speech ability. Except for the thin splenium of corpus callosum, the neurologic imaging was also unremarkable (Huijmans et al., 2017). Therefore, there was not enough evidence to support the pathogenesis of *MOCS3* variation and studies illustrating the phenotypic spectrum of *MOCS3-*induced Moco deficiency are needed.

Here we described a case of Moco deficiency identified with compound heterozygous variations in the *MOCS3* gene. The infant presented aggressive phenotypes of Moco deficiency including early onset of neonatal seizures, hypertonia, feeding difficulty, and severe developmental delay. The MRI indicated global cystic encephalomalacia and cortical necrosis. The patient showed poor reaction to treatment and resulted in infant death. To the best of our knowledge, our report is probably the first study to prove that *MOCS3* is a pathogenic gene of Moco deficiency. Our study may contribute to genetic diagnosis of Moco deficiency and future genetic counseling.

## Case Description

Our patient is the second child born to the non-consanguineous Chinese parents. The first male child presented neonatal seizures, hypotonia, and developmental delay. He died at the age of 8 months and the suspected diagnosis was hypoxic-ischemic encephalopathy, which was made by Magnetic resonance imaging (MRI) presentation.

**Figure 1:**
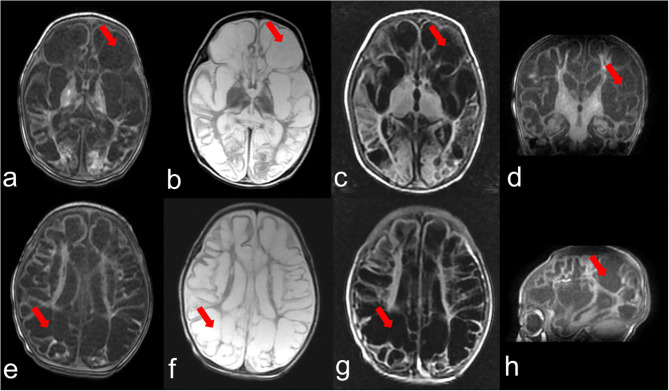
Brain MRI at the age of 3 months. **(a–c)** represent T1,T2,T2-FLAIR weighted images of one section and **(e–g)** indicate T1,T2,T2-FLAIR weighted images of another section, respectively. **(d,h)** show T1 weighted images of the coronal plane and sagittal plane. The arrows point out the representing lesions in each picture.

The second female child was born at full term (40 weeks) with no abnormality in pregnancy. Without any inducement, generialized tonic-clonic seizures were observed within 24 h after birth and seizures were not controlled after multiple treatments. Her after birth physical examination indicated microcephaly [occipitofrontal head circumference 30.0 cm, < −3 Standard deviation (SD)], hypotonia, and hyper-reflexia. At the age of 5 months the patient was admitted to hospital for the reoccurrence of the seizures. She did not develop the ability to lift her head and trace. MRI showed frontotemporal, parietal, and occipital lobes cystic encephalomalacia and bilateral basal ganglia atrophy (Figure 1). The patient showed severe neurodevelopmental delay. She was still unable to trace, lift her head, roll over, and sit at 10 months. The patient had feeding difficulties resulting in low breast milk intake and died at age of 12 months because of lung infection.

## Diagnostic Assessment

**Figure 2:**
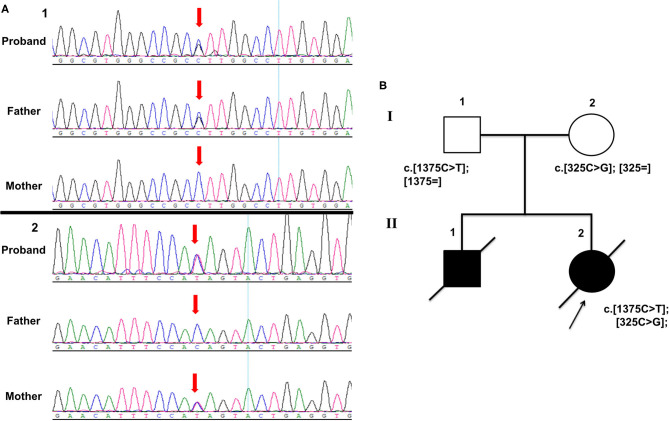
**(A)** Sanger sequencing electropherograms showing compound heterozygous variants in *MOCS3* (**A** showing c.1375C>T and **B** showing c.325C>G). **(B)** Pedigree chart of the family.

Whole-exome sequencing (WES) accompanied with Sanger sequencing validation was performed in the proband and her parents (Figure 2). The variants were firstly filtered according to HGMD and ACMG guidelines Disease-causing mutations (DM) and probable/possible pathological mutation (DM) in the HGMD (Professional version 2019.1) database, and pathogenic (P) and likely pathogenic (LP) variants interpreted by ACMG guidelines for interpretation of genetic variants were included. Secondly, variants were filtered according to allele frequency, variant type, and inheritance mode. Variants with minor allele frequencies (MAF) <0.1%, variant depth of coverage ≥20 and alteration base depth of coverage ≥4 were taken on for further analyses. Filtering was conducted on the remaining variants according to variant type and inheritance model of the associated disease. The proband was identified with compound heterozygous variants in NM_014484.49 (*MOCS3*): c.1375C>T; p.Gln459Ter (chr20:49576754-49576754) and NM_014484.49 (*MOCS3*): c.325C>G; p.Leu109Val (chr20:49575704-49575704). Her father was a heterozygous carrier of the *MOCS3* c.1375C>T variant and the mother carried another variant in the *MOCS3* c.325C>G. All the segregated variants of this case were listed in the Supplementary Table 1 and the annotation of each variant is given.

The variants are evaluated by MutationTaster (v2.0), CADD (v1.6), FATHMM (v2.3). The scores of each variant of *MOCS3* from the bioinformatic prediction tools are as follows: for c.325C>G the scores are 1.000, 22.8, and 1.19 and for c.1375C>T (premature termination mutation) the scores are 0.958, 36, NA, respectively. The allele frequency of c.325C>G was 6.181e−05 in gnomAD database, 5.182e−05 in the ExAC and 0 in the 1,000 genomes database. The allele frequency of c.1375C>T was 0 in gnomAD the ExAC and the 1,000 genomes databases.

Moco deficiency-related laboratory tests were conducted. The urine and plasma uric acid levels were detected by Beckman Coulter AU680 and enzymatic colorimetric assay on the Cobas 8000 system. The urine sulfite was tested by a urine sulfite test strip. The urine uric acid level was 32 mmol/mol creatinine (normal range 350–2,500 mmol/mol creatinine) and plasma uric acid level was 0.01 mmol/l (normal range 0.08–0.37 mmol/l). The urine sulfite test strip was positive.

## Discussion

Moco deficiency is characterized by severe neonatal neuropathologic symptoms, typical neuroimaging findings and variants in causative genes *MOCS1, MOCS2*, and *GPHN* (Atwal and Scaglia, 2016). These genes along with *MOCS3* are involved in Moco biosynthesis and providing cofactors to Moco dependent enzymes. In reported Moco deficiency cases, two thirds are caused by *MOCS1* variants, followed by *MOCS2* and *GPHN*. Most of these cases presented typical symptoms of Moco deficiency (Reiss and Johnson, 2003; Zaki et al., 2016; Mayr et al., 2021). Until now there was no evidence that *MOCS3* was one of the causative genes of Moco deficiency. In 2017 there was one case with the *MOCS3* variant who showed a slight disorder of sulfite metabolites and near to normal neurologic symptoms (Huijmans et al., 2017). In our study, we identified a Moco deficiency patient with variants in *MOCS3* presented typical features of Moco deficiency including early onset severe neurological symptoms accompanied with typical MRI manifestations and biochemical changes. The published variant in 2017 was a missense variant and the variants in our study included a premature termination variation, and it could be a possible reason for the severity of our case. To our knowledge, our study is the first study to show that *MOCS3* is a pathogenic gene of Moco deficiency.

The clinical diagnosis of Moco deficiency is often supported by typical MRI readings (brain edema, cystic encephalomalacia, atrophy of cortex and white matter, focal or bilateral changes of globus pallidus, thalamus, etc.), and it has to be confirmed by genetic and biochemical testing (Durmaz and Ozbakir, 2018; Arican et al., 2019). The primary features of the disease were reported to be seizures (72%), feeding difficulties (26%), hypotonia (11%), and developmental delay (9%). The median onset age was the first day of life and the median survival was 36 months (Mechler et al., 2015). In our study, the proband presented similar symptoms as the first infant, and the inherited diseases were considered and further tests were performed. Although the first infant was suspected as hypoxic-ischemic encephalopathy, in some cases the neurological manifestations of Moco deficiency may overlap with severe hypoxic-ischemic encephalopathy, and shows seizures, developmental delay, and cystic encephalomalacia in MRI etc. (Zaki et al., 2016; Yoganathan et al., 2018). However, Moco deficiency usually presents more aggressive neurological symptoms. The positive urine test for sulfite with decreased plasma and urine uric acid would assist diagnosis (Yoganathan et al., 2018; Bender et al., 2019).

MOCS3 might involve in the pathogenesis of neurodevelopmental diseases by impacting the Moco biosynthesis pathway, affecting neuronal receptors and causing neuronal death. *MOCS3* knocked out cells were reported to cause combined deficiency of sulfite oxidase, xanthine oxidoreductase, and aldehyde oxidase (Chowdhury et al., 2012). This resulted in accumulation of many metabolites, especially sulfite, a neurotoxin proved to react with cystin to form the S-sulfocysteine and thiosulfate (Neukranz et al., 2019). The structure of S-sulfocysteine highly resembled the excitatory neurotransmitter glutamate that could bind and stimulate glutamatergic receptors. It has been proved in a zebrafish model that S-sulfocysteine could stimulate glutamatergic receptors, induce seizure-like movements, and increase cell death in the central nervous system (Zaki et al., 2016; Plate et al., 2019). Accumulation of S-sulphocysteine and thiosulfate would activate the N-methyl-D-aspartate receptor and increase intracellular magnesium and calcium. This could enhance the permeability of the mitochondria, compromise the mitochondrial energy supply, and reduce the cell viability in cerebral cortex of rat brain (Grings et al., 2014; Marelja et al., 2018; Bender et al., 2019; Plate et al., 2019). All these processes may be the possible mechanisms underlying the neurological dysfunction observed in our patient.

In summary, we reported a novel causative gene *MOCS3* in a Moco deficiency patient. Our study may contribute to genetic diagnosis of Moco deficiency and future genetic counseling.

## Materials and Methods

Leukocyte DNA was extracted from peripheral blood using the phenol-chloroform method. DNA was then sheared to ~200 bp by the Biorupter UCD-200 (Diagenode). The DNA fragments were then repaired at the end, and one A base was added to the 3′ end. The DNA fragments were connected with sequencing adaptors, and fragments of ~320 bp were collected by XP beads. After PCR amplification, the DNA fragments were hybridized and captured by IDT's xGen Exome Research Panel (Integrated DNA Technologies, San Diego, USA) according to the manufacturer's protocol. The hybrid products were eluted and collected. Then, DNA was PCR amplified and purified. The libraries were tested for enrichment by qPCR, and size distribution and concentration were determined using an Agilent Bioanalyzer 2100 (Agilent Technologies, Santa Clara, CA, USA). WES was performed on the HiSeq 2500 system (Illumina) with an average coverage depth of 100 × of the variants for sequencing the genomic DNA of the family. Raw sequence reads were mapped to the hg19/GRCh37 using Elandv2e (CASAVA1.8.2; Illumina) to remove duplicated reads and CASAVA1.8.2 was further used to call single-nucleotide variants and short insertions/deletions (Markus et al., 2020; Tessarech et al., 2020). The variants were annotated using ANNOVAR (Wang et al., 2010).

A public database (1,000 Genomes Project, ExAC, gnomAD) was used to detect variants frequencies. The pathogenicity of variants was predicted using following software programs:

Combined Annotation Dependent Depletion [CADD] [https://cadd.gs.washington.edu/] (Kircher et al., 2014; Rentzsch et al., 2019).

MutationTaster [http://www.mutationtaster.org/] (Schwarz et al., 2014).

FATHMM [http://fathmm.biocompute.org.uk/fathmmMKL.htm] (Shihab et al., 2013a,b).

Sanger sequencing was performed on the DNA of the proband's parents to validate the variants found in WES using standard methods on ABI 3730 automated sequencer with BigDye™ Terminator v3.1 Cycle Sequencing Kit, as described previously (Stockley et al., 2013). The primers are as follows: forward primer (5′-CTCTGTCCCGAGATGAGATTCT-3′) and reverse primer (5′-CGTTGTCCGAGCAGTCAGC-3'), forward primer (5′-AAGAAGCAATCTGGGAAGAGAAG) and reverse primer (5′-ATGACCCTCTTTCTGAATAATTAAA).

## Data Availability Statement

The original contributions presented in the study are included in the article/Supplementary Material, further inquiries can be directed to the corresponding author/s.

## Ethics Statement

The study was approved by the Ethics Committee of the Maternal and Child Health Hospital of Hunan Province (2020-S003). Informed consent was obtained from the legal guardian of the participant for the publication of this case report (including all data and images).

## Author Contributions

XM and HW designed the research. QT and YaC interpreted the data and wrote the manuscript. HW, LS, YoC, YW, and YuC did the follow-up study and collected, evaluated the clinical, and genetic evidence. QT and HW revised the manuscript. All authors read and approved the final manuscript.

## Conflict of Interest

The authors declare that the research was conducted in the absence of any commercial or financial relationships that could be construed as a potential conflict of interest.
